# P15 Direct provocation test for penicillin allergy delabelling: a 4-year retrospective review

**DOI:** 10.1093/jacamr/dlad066.019

**Published:** 2023-06-14

**Authors:** Ellen Sugrue, Edward D H McKee, Rebecca K Sutherland

**Affiliations:** Regional Infectious Diseases Unit, Western General Hospital, Edinburgh, Scotland; Regional Infectious Diseases Unit, Western General Hospital, Edinburgh, Scotland; Regional Infectious Diseases Unit, Western General Hospital, Edinburgh, Scotland

## Abstract

**Background:**

Penicillin allergy is the most commonly reported drug allergy.^1^ Over 90% of these are mislabelled^1^. Labelled penicillin allergy is associated with increased broad-spectrum antibiotic use, increased antibiotic costs, and increased length of admission. A recent meta-analysis of delabelling studies showed that drug provocation test (DPT) is a low-risk procedure in patients with multiple clinical and financial benefits.^2^

**Methods:**

Retrospective analysis of all patients who had DPT in hospital across one health trust 2019–2022. Hospital and GP electronic records of drug allergies were reviewed. GPs of patients who retained an allergy status post-DPT were contacted.

**Results:**

46 DPTs were documented during this time. The PENFAST^3^ score was used to identify those suitable for DPT. One delayed reaction was recorded. No anaphylaxis was recorded. Infectious diseases were the primary specialty for DPT (67%). In total, 78% were carried out on medical patients. ID consult service was responsible for 100% of DPTs in surgical specialties. Seven patients who received DPT had been relabelled penicillin allergic post-DPT. Two were self-reported on presentation to hospital. Five were from physician review of historic documentation in hospital settings. In total, 56% of patients had not had GP documentation regarding allergy status updated.

**Conclusions:**

This review concludes that DPT is safe in an inpatient population and inpatient review of reported penicillin allergy is an opportunity to identify mislabelled allergy. Infection specialists were the primary teams for DPT however given the safety profile of the DPT non-infection, and allergy specialists should be encouraged to use the protocol. Enhanced communication is needed with GPs to ensure the correct status is known in the community. Relabelling of penicillin allergy is a risk, and further education must be given to patients and healthcare providers post-DPT to ensure continuation of delabelled status.
